# Single-stage internal fixation with knee arthroplasty: a novel approach for concurrent knee osteoarthritis and distal femoral fracture

**DOI:** 10.1093/jscr/rjaf322

**Published:** 2025-05-22

**Authors:** Yinping Liu, Guosong Xu

**Affiliations:** Department of Orthopedics, The First Hospital of Putian City, Putian City, Fujian Province, China; Department of Orthopedics, The First Hospital of Putian City, Putian City, Fujian Province, China

**Keywords:** total knee arthroplasty, distal femoral fracture, osteoarthritis, single-stage surgery, rehabilitation

## Abstract

This study investigates the clinical outcomes of single-stage internal fixation combined with total knee arthroplasty (TKA) for treating distal femoral fractures with concurrent knee osteoarthritis (OA). We report a case of a 68-year-old female patient who sustained a displaced distal femoral spiral fracture (AO/OTA type A1.2) and severe knee OA (Kellgren-Lawrence grade IV). The patient underwent single-stage surgery involving locked compression plating for fracture fixation and primary TKA. Postoperatively, the patient was allowed immediate weight-bearing and aggressive rehabilitation. At 4-year follow-up, the patient demonstrated significant functional improvement, with an Oxford Knee Score of 36.8 and a visual analog scale (VAS) pain score of 1.9. Radiographs showed no implant loosening and normal knee alignment. This case highlights the feasibility and effectiveness of single-stage internal fixation combined with TKA for elderly patients with osteoporotic distal femoral fractures and knee OA, offering a promising treatment option for such complex cases.

## Introduction

Distal femoral fractures in the elderly are increasingly prevalent due to the rising incidence of osteoporosis and age-related falls. Managing these fractures is challenging when concurrent knee osteoarthritis (OA) exists, as it demands both fracture stabilization and joint restoration. Traditional open reduction and internal fixation (ORIF) for distal femoral fractures in osteoporotic bone carries risks of nonunion, malunion, and worsening OA. Total knee arthroplasty (TKA) has emerged as an alternative, offering pain relief, improved function, and accelerated rehabilitation compared to ORIF. However, concerns remain regarding implant stability, infection risk, and prosthesis selection. Single-stage internal fixation combined with TKA represents an innovative approach, addressing both fracture and arthritis in one procedure, potentially leading to faster recovery and improved outcomes. While some studies have explored this approach, limited data exists, particularly regarding long-term outcomes and specific patient selection criteria. This case report demonstrates the feasibility and efficacy of single-stage internal fixation combined with TKA for an elderly patient with a distal femoral fracture and severe knee OA. We will discuss the potential advantages and limitations of this approach and review the relevant literature.

## Case presentation

**Figure 1:**
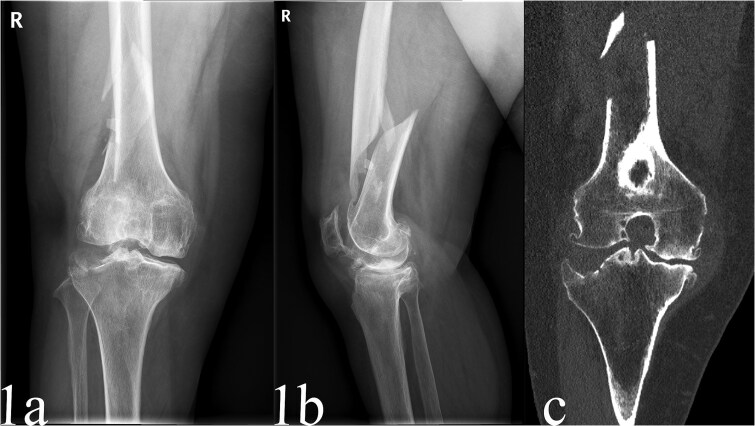
Preoperative anteroposterior radiographs of the case, sustained a displaced spiral fracture of the distal femur (AO/OTA type A1.2) and knee OA (Kellgren-Lawrence grade IV) (a and b). CT scans knee OA (Kellgren-Lawrence grade IV) (c).

A 68-year-old female with a history of advanced tricompartmental osteoarthritis presented with persistent knee pain and functional impairment following a fall at home. Her OA had been managed conservatively with analgesics and intra-articular injections, but her pain had progressively worsened, impacting her well-being and activities of daily living. Imaging revealed a displaced spiral fracture of the distal femur (AO/OTA type A1.2) and knee OA (Kellgren-Lawrence grade IV) (Fig. 1a and b). Computed tomography (CT) scans further clarified the fracture pattern and cartilage degeneration (Fig. 1c). Laboratory tests were unremarkable except for mild anemia, and bone density assessments confirmed osteoporosis (T-score of −2.8). After thorough evaluation and discussion with the patient regarding treatment options, a single-stage procedure combining ORIF of the femoral fracture with TKA was chosen.

### Surgical technique

**Figure 2:**
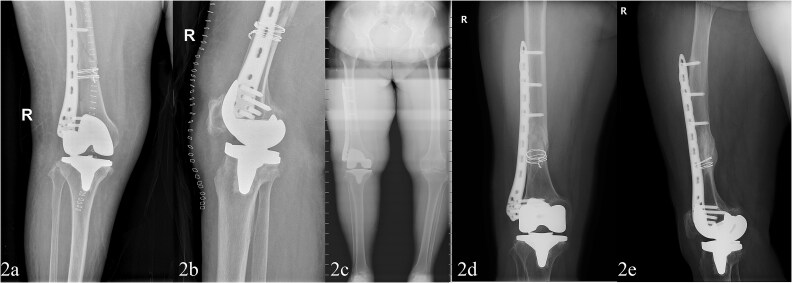
Post-operative fracture fixation with TKA and locking plate (a and b). Four years later, healed for review only fracture with implants in situ (c–e).

The procedure was performed under spinal anesthesia with cefazolin and tranexamic acid administered for infection prophylaxis and bleeding control. A midline anterior knee incision was made to expose the joint and fracture site. Intraoperatively, a spiral fracture and full-thickness cartilage wear were found. The surgical steps included: (i) provisional fracture reduction with cerclage wires; (ii) performing necessary tibial, femoral, and patellar cuts and trial reductions (Femoral alignment was achieved using an intramedullary rod inserted after provisional fracture stabilization with bone clamps and cerclage wires); (iii) TKA using a standard cruciate-sacrificing prosthesis; (iv) application of a locked compression plate for fracture fixation; (v) wound closure after stability testing. The TKA prosthesis used was the Attune primary TKA system (DePuy Synthes). The procedure lasted 124 min with an estimated blood loss of 105 ml. Immediate post-operative imaging is shown in Fig. 2a and b.

Femoral alignment was achieved using an intramedullary rod inserted after provisional fracture stabilization with bone clamps and Kirschner wires. Fracture reduction preceded rod placement to ensure anatomical restoration prior to definitive bone resection.

### Post-operative protocol

Postoperatively, the patient was initiated on a multimodal pain management plan and aggressive physical therapy. Considering the stable fixation achieved during the surgery and the importance of early weight-bearing, Full weight-bearing as tolerated was initiated immediately with assistive devices and range of motion exercises were gradually introduced.

### Assessment of outcomes

The patient resumed light housework at 8 weeks and walked independently without aids at 12 weeks. At the final follow-up, 4 years post-surgery, the patient demonstrated substantial improvement. The Oxford Knee Score increased to 36.8, the VAS pain score decreased to 1.9, and radiographs showed no implant loosening and normal knee alignment (Fig. 2c–e). The patient regained independent ambulation and had an improved quality of life.

## Discussion

Managing distal femur fractures in elderly patients with osteoporosis and arthritis is challenging [1]. This case demonstrates the feasibility of single-stage internal fixation combined with primary TKA, allowing immediate weight-bearing post-surgery to accelerate recovery.

TKA is increasingly used for such fractures, as traditional ORIF may lead to nonunion, malunion, and secondary arthritis in osteoporotic bone [1–3]. TKA addresses both fracture and arthritis, enabling early mobilization, and weight-bearing, crucial for reducing systemic complications from prolonged inactivity in the elderly. Recent studies show good long-term outcomes for TKA in native knee fractures [3]. However, these case series often utilize special prostheses, including constrained designs, long-stem components, and even modular tumor prostheses. Increased constraint provides greater stability for the implant but at the cost of increased forces transmitted to the implant interface and potential subsequent aseptic loosening [4]. Non-constrained standard prostheses are only suitable for simple fracture types and patients with sufficient bone stock and are unable to address the issue of fracture stability.

To avoid the drawbacks of specialized constrained prostheses, the combined application of internal fixation and TKA for certain fracture types, although rarely reported, presents a viable alternative. Choi *et al.* used a medial pivot knee prosthesis and internal fixation with screws or titanium cables for eight patients, achieving good results in 15 weeks, but they required external fixation for additional support and were unable to fully weight-bearing in the early stage [5]. Antao *et al.* used a bifold fixation and total knee arthroplasty, with cost-effective and positive results [6]. Robles *et al.* combined buttress plating with constrained condylar knee arthroplasty for a patient, getting good results [7]. Chen *et al.* noted that Type A fractures could be managed with long-stemmed femoral components, necessitating limited internal fixation to enhance stability for better function and weight-bearing [8].

In our case, an extended-stem TKA alone couldn’t handle the spiral fracture’s rotational instability. Combining internal fixation and TKA led to better function and faster weight-bearing. Patient selection is key. Comorbidities, life expectancy, activity level, bone quality, and rehabilitation support all matter. The surgical technique is also crucial. We first stabilized the fracture with bone clamps or wires, then performed femoral reaming and implant insertion, and finally used a Less Invasive Stabilization System (LISS) plate for added stability, enabling early mobility.

Despite positive results, this approach has drawbacks. It’s more traumatic than simple internal fixation, and preoperative assessment is needed to minimize risks. Longer surgery may increase infection risk, though none occurred in our case. Overall, single-stage internal fixation with standard TKA is promising for selected elderly patients but it should be performed in specialized centers. Future research should focus on refining patient selection, optimizing surgical techniques, and improving implant design. Larger-scale, preferably randomized controlled trials, and long-term follow-up studies are needed to confirm its superiority.

## References

[1] Moloney GB, Pan T, Van Eck CF, et al. Geriatric distal femur fracture: are we underestimating the rate of local and systemic complications? Injury 2016;47:1732–6. 10.1016/j.injury.2016.05.024 27311551

[2] Tampere T, Ollivier M, Jacquet C, et al. Knee arthroplasty for acute fractures around the knee. EFORT Open Rev 2020;5:713–23. 10.1302/2058-5241.5.190059 33204515 PMC7608576

[3] Aebischer AS, Hau R, de Steiger RN, et al. Distal femoral arthroplasty for native knee fractures: results from the Australian Orthopaedic Association National Joint Replacement Registry. Bone Joint J 2022;104-B:894–901. 10.1302/0301-620X.104B7.BJJ-2021-1136.R3 35775178

[4] Zhang J, Li E, Zhang Y. Prostheses option in revision total knee arthroplasty, from the bench to the bedside: (1) basic science and principles. EFORT Open Rev 2022;7:174–87. 10.1530/EOR-21-0089 35192509 PMC8897564

[5] Choi NY, Sohn JM, Cho SG, et al. Primary total knee arthroplasty for simple distal femoral fractures in elderly patients with knee osteoarthritis. Knee Surg Relat Res 2013;25:141–6. 10.5792/ksrr.2013.25.3.141 24032103 PMC3767900

[6] Antao NA, Londhe S, Toor R, et al. Short-term results of a novel management of supracondylar fracture with coexisting osteoarthritis with bifold fixation and total knee arthroplasty. Art Ther 2021;3:44. 10.1186/s42836-021-00098-0 PMC8796440 35236499

[7] Robles EL, Carbone JJ, Farr J. Management of a distal femoral nonunion in a patient with a failed unicompartmental knee arthroplasty: a case report. J Knee Surg 2023;36:1071–6.

[8] Chen CH, Chen CH, Tsai CL, et al. Total knee arthroplasty with a long-stem femoral component for the treatment of distal femoral fractures. J Trauma Acute Care Surg 2017;82:1063–7.28520687

